# Histological and Immunohistochemical Features of the Skin Lesions in CANDLE Syndrome

**DOI:** 10.1186/1546-0096-13-S1-P154

**Published:** 2015-09-28

**Authors:** A Torrelo, I Colmenero, L Requena, A Paller, Y Ramot, C-CR Lee, A Vera, A Zlotogorski, R Goldbach-Mansky, H Kutzner

**Affiliations:** 1 Hospital Niño Jesús, Dermatology, Madrid, Spain; 2 Children's Hospital, Pathology, Birmingham, UK; 3 Fundación Jiménez Díaz, Dermatology, Madrid, Spain; 4 Northwestern University, Dermatology, Chicago, IL, USA; 5 Hadassah-Hebrew University Medical Center, Dermatology, Jerusalem, Israel; 6 NIH, Pathology, Bethesda, MD, USA; 7 Hospital Carlos Haya, Dermatology, Málaga, Spain; 8 NIH, Transalational Autoinflammatory Disease Section, Bethesda, MD, USA; 9 Dermatohistopathologisches Gemeinschaftslabor, Pathology, Friedrichshafen, Germany

## Question

Chronic atypical neutrophilic dermatosis with lipodystrophy and elevated temperature (CANDLE) syndrome is caused by mutations in *PSMB8*. It occurs with early-onset fevers, accompanied by a widespread, violaceous and often annular, cutaneous eruption. It is postulated that the inflammatory disease manifestations stem from excess secretion of interferons, mostly type I interferons, which are proposed to lead to the recruitment of immature myeloid cells into the dermis and subcutis.

## Methods

We systematically analyzed skin biopsies from 6 CANDLE syndrome patients by routine histopathology and immunohistochemistry methods.

## Results

In all cases, skin lesions showed the presence of extensive mixed dermal and subcutaneous inflammatory infiltrate, composed of mononuclear cells, atypical myeloid cells, neutrophils, eosinophils and some mature lymphocytes. Positive LEDER and myeloperoxidase staining supported the presence of myeloid cells. Positive CD68/PMG1 and CD163 staining confirmed the existence of histiocytes and monocytic macrophages in the inflammatory infiltrate. CD123 staining was positive, demonstrating the presence of plasmacytoid dendritic cells.

## Conclusion

The histopathology and IHC panel in the skin lesions of CANDLE syndrome is highly specific and should lead to a promto and specific diagnosis of this disorder. Both histopathology and IHC provide further insight into the pathogenesis of CANDLE syndrome.

